# Management of Discolored Failure Root Canal-Treated Upper Lateral Incisor

**DOI:** 10.1155/2020/8202873

**Published:** 2020-05-26

**Authors:** Nik Abdul Ghani Nik Rozainah, Azih Nurul Farah, Mohmed Isaqali Karobari

**Affiliations:** ^1^Conservative Department, School of Dental Sciences, Health Campus, Universiti Sains Malaysia, 16150 Kubang Kerian, Kelantan, Malaysia; ^2^Conservative Unit, School of Dental Sciences, Health Campus, Universiti Sains Malaysia, 16150 Kubang Kerian, Kelantan, Malaysia

## Abstract

Root canal treatment failure can be determined based on a patient's complaint and on the basis of clinical examination and radiographic findings. Most of the signs and symptoms for the failure are pain and discomfort, swelling and sinus formation at the surrounding soft tissue, and discoloration of the subjected tooth. Factors such as mechanical perforation during the procedures, overfilled or underfilled root canal, and missed or unfilled canals are the main factors for the failure outcome. This case report presents a discolored and infected upper lateral incisor which was previously root canal treated. The tooth was successfully managed under nonsurgical and surgical retreatment followed by an internal bleaching and full porcelain veneer. Apical tissue healing and acceptable tooth appearance was observed during a 12-month review.

## 1. Introduction

The successful outcome of root canal treatment is highly dependent on the elimination of persistent infection and reinfection of the root canal space. Failure can be determined on the patient's complaint, during basic clinical examination, and from radiographic findings. It is more related with the lack of knowledge on the part of the operator and lack of referral for the complex anatomy cases and severe root curvature to be treated by a specialist [1]. Many studies have shown that the failures were related to the presence of extraradicular biofilms, root fracture, mechanical perforations, residual necrotic pulp tissue, broken instruments, periodontal disease, root canal overfilling, root canal underfilling, missed canals or unfilled canals, and insufficient coronal restoration after completion of root canal treatment [2–4]. This clinical report presents a young man who had experienced a root canal treatment failure of his upper left lateral incisor. The tooth had been poorly treated, where the extruded root canal filling had caused a continuous and persistent periapical lesion. At the same time, poorly obturated material had contributed to the tooth discoloration. Root canal retreatment was successfully constructed for the tooth followed by internal bleaching and E-max full porcelain veneer. The successful outcome was observed after a year, where there was evidence of periapical healing and a satisfactory appearance of the tooth.

## 2. Case Presentation

**Figure 1:**
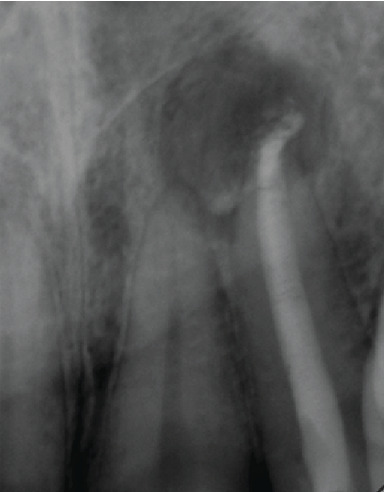
Periapical radiograph showing that tooth 22 had a well-condensed root canal filling, where the gutta percha was overextended with a periapical lesion at the area.

**Figure 2:**
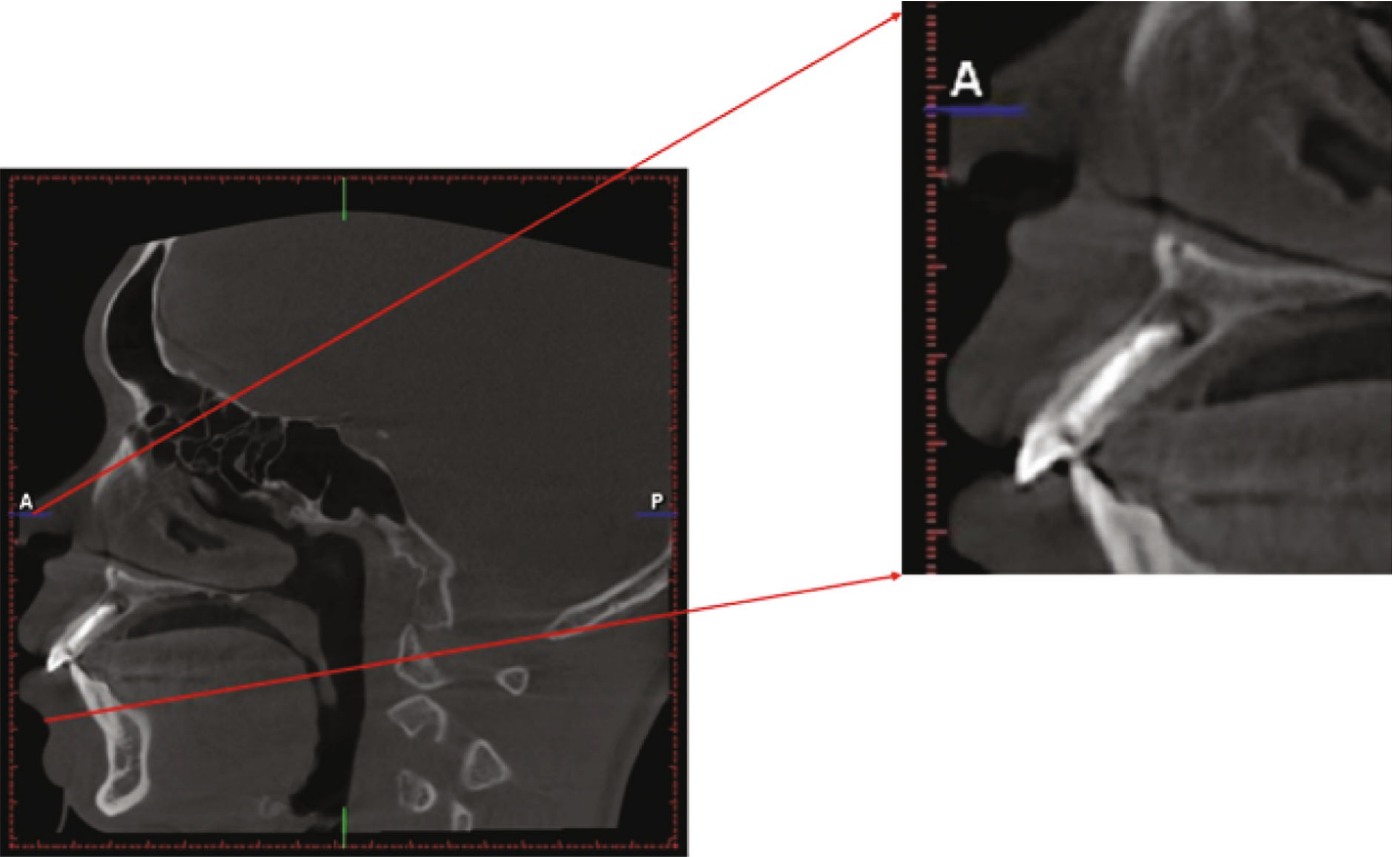
Cone Beam Computed Tomography (CBCT) of tooth 22 with a periapical lesion.

A 26-year-old man was referred to the Conservative Clinic for the management of the discoloration of a previous root canal treatment for tooth 22. The patient claimed to have had a history of trauma during his teenage years, and the tooth had multiple episodes of pain and swelling. The lesion healed after the completion of root canal treatment which was almost 5 years back. However, for the last few months, the tooth started to cause discomfort with a slight pain during biting or chewing. He also noticed that it became darker and blackish in color. The patient otherwise was cleared from any medical illness, and his health condition was fit and healthy. During an intraoral examination, tooth 22 showed dark discoloration and was tender to palpation and percussion. A 1 mm × 1 mm soft tissue swelling was located at the palatal site of the tooth. Thermal test and electronic pulp test indicated a positive response of the adjacent teeth 21 and 23. Periapical radiograph showed that tooth 22 had a well-condensed root canal filling; however, the gutta percha was overextended and there was periapical lesion at the area (Figure 1). Cone Beam Computed Tomography (CBCT) revealed that the size of the lesion was approximately 2 mm × 2 mm (Figure 2). The provisional diagnosis was a failure root canal-treated tooth with chronic apical periodontitis and discolored tooth. After discussion and explanation, the patient agreed for nonsurgical retreatment to remove the infection and extruded gutta percha, thus eliminating and healing the apical periodontitis before undergoing an internal bleaching. The patient was also aware of the possibility of periapical surgery as a complementary approach to resolve the pathology if the conventional retreatment failed to succeed.

### 2.1. Nonsurgical Root Canal Retreatment

**Figure 3:**
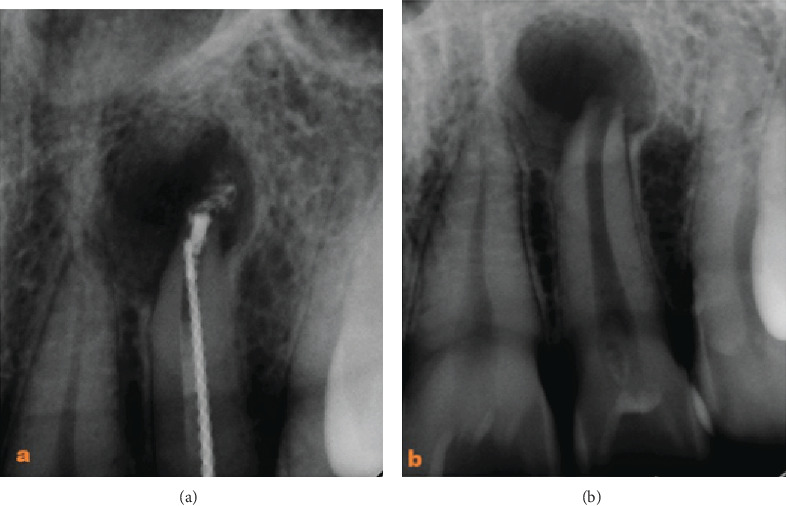
Periapical radiograph of tooth 22 after GP removal with a Hedstrom file.

During retreatment, a Dental Operating Microscope (Zeiss OPMI ® pico, Germany) was used to enhance illumination and magnification to reach the apical root and assess the extruded gutta percha. The gutta percha was removed with the use of a chloroform solvent (Sultan Healthcare Inc., USA). Apical gutta percha was hooked and removed with the Hedstrom file #40 and #45 (DiaDent, South Korea) (Figure 3). The canal was rinsed with sodium hypochlorite 2.5% (HUSM Pharmacy, Malaysia) and a new corrected working length was confirmed. No canal preparation was done since it was already shown to have an acceptable diameter and was tapered; furthermore, the apical end showed some root resorption and loss of apical stopper. Nonsetting calcium hydroxide paste (Pulpdent, USA) intracanal medicament was placed into the canal, and the coronal seal was achieved by Fuji IX Glass Ionomer Cement (GC International, Japan). The patient came for a review after 4 weeks; the palatal swelling was completely healed, and he also denied any pain. However, radiographically, the size of the apical lesion remained the same. We discussed with the patient regarding periapical surgery to resolve the pathology and for histopathology biopsy. Verbal and written consent was obtained from the patient. Obturation was performed with F3 ProTaper Gutta Percha (Dentsply Maillefer, USA) as a filling material together with AH plus sealer (Dentsply Maillefer, USA). The root canal was filled up to the new working length 2 mm short from the open apex. The cavity access was sealed with the composite resin Zmack microhybrid (Zhermack SpA, Italy) for a permanent coronal seal.

### 2.2. Periapical Surgery

**Figure 4:**
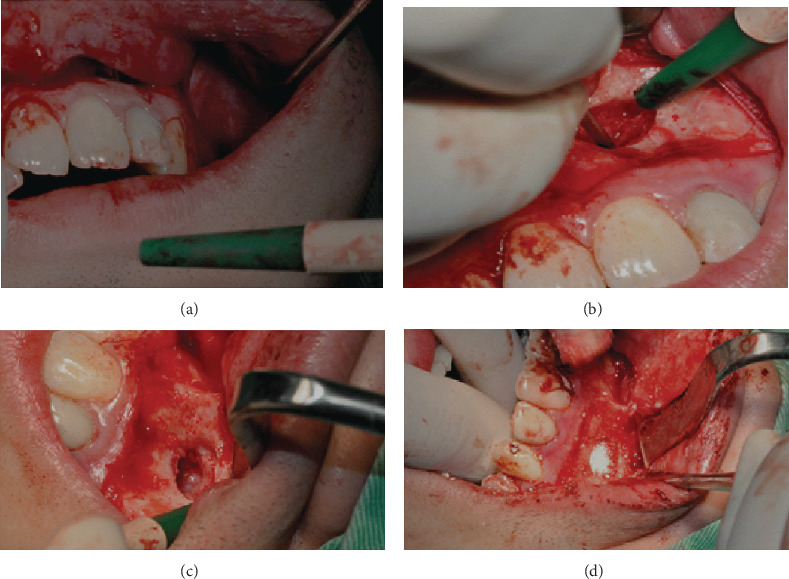
Periapical surgery procedure of tooth 22: (a) flap preparation, (b) enucleation of lesion, (c) bone preparation for graft, and (d) bone graft placement.

**Figure 5:**
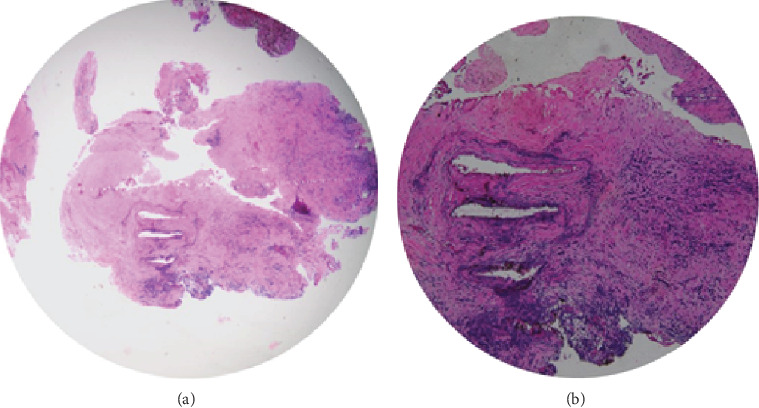
Histopathological finding of lesion: (a) collagenous tissue with mature fibroblasts, patchy chronic inflammatory cells, scanty vascular channels, and nerve bundle; (b) loosely edematous background with admixed hemorrhage, numerous capillaries, and intense inflammatory cells comprising lymphoplasma cells, macrophages, and neutrophils.

During periapical surgery, the operating site was anesthetized with 2% lignocaine containing 1 : 100,000 adrenaline. The Luebke-Oschenbein flap was designed, and once the flap was raised, there was a buccal bone resorption which presented as a small hole measuring 1 mm × 1 mm. Once the lesion was excised, the exposed root tip with an open apex was filled with Mineral Trioxide Aggregate (ProRoot® MTA, Dentsply Sirona, Canada). Freeze-dried cancellous Osteo-Lamb bone graft (USM Tissue Bank, Malaysia) was placed at the bony defect to encourage the growth of key surrounding tissue (Figure 4). Flap closure was carried out with 3-0 VICRYL suture (Ethicon, USA). Oral intake of ibuprofen 600 mg was prescribed when necessary. Histopathological examination revealed fragments of densely collagenous tissue with mature fibroblasts, patchy chronic inflammatory cells, scanty vascular channels, and nerve bundles. A focal area shows a loosely edematous background with admixed hemorrhage, numerous capillaries, and intense inflammatory cells comprising lymphoplasma cells, macrophages, and neutrophils (Figure 5). No epithelial cell lining was observed. The definitive diagnosis was periapical granuloma.

### 2.3. Nonvital Bleaching

**Figure 6:**
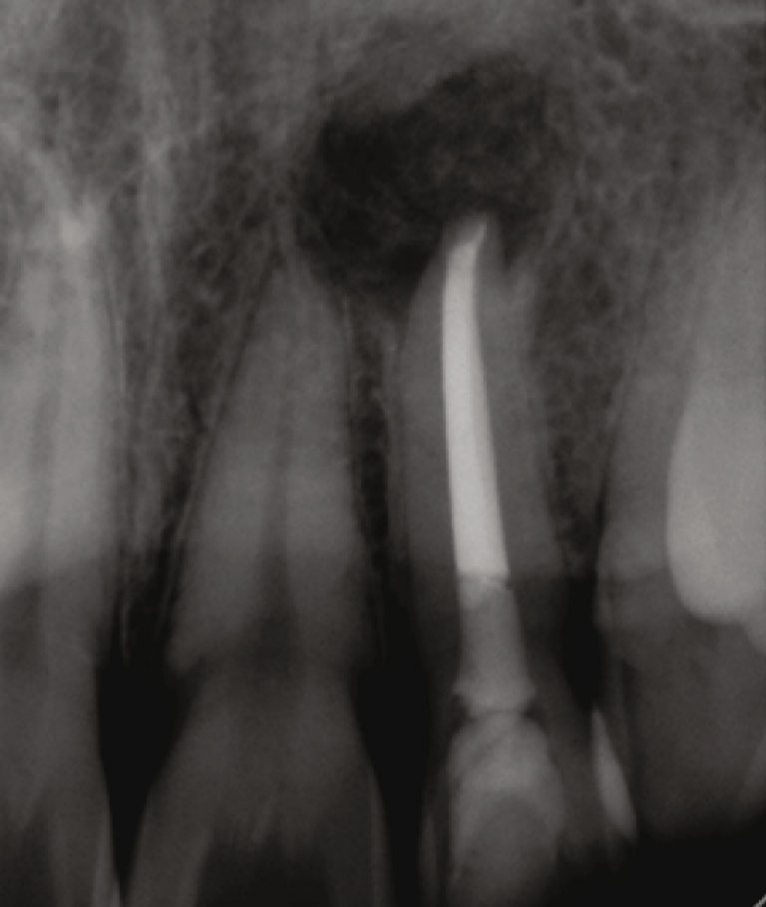
Posttreatment radiograph after 6 months.

During 6 months of review, the healing was successful and the patient denied any complaint (Figure 6). The tooth was prepared for an internal bleaching under rubber dam isolation. During the procedure, 2 mm gutta percha was cut in the apical direction beyond the cemento-enamel junction. The area was then washed with normal saline, rinsed, and dried. A thin layer of 1 mm Glass Ionomer Fuji II (GC International, Japan) was placed to cover the gutta percha prior to the application of 35% hydrogen peroxide agent (Ultradent Opalescence™ Endo, USA). The bleaching agent was expressed into the open pulp chamber. The cavity was temporarily filled using GC Fuji IX glass ionomer cement (GC International, Japan). The procedure was repeated for every three continuous follow-up visits in three weeks' time until desired results were obtained. Composite resin Zmack microhybrid (Zhermack SpA, Italy) was used as a final and permanent filling.

**Figure 7:**
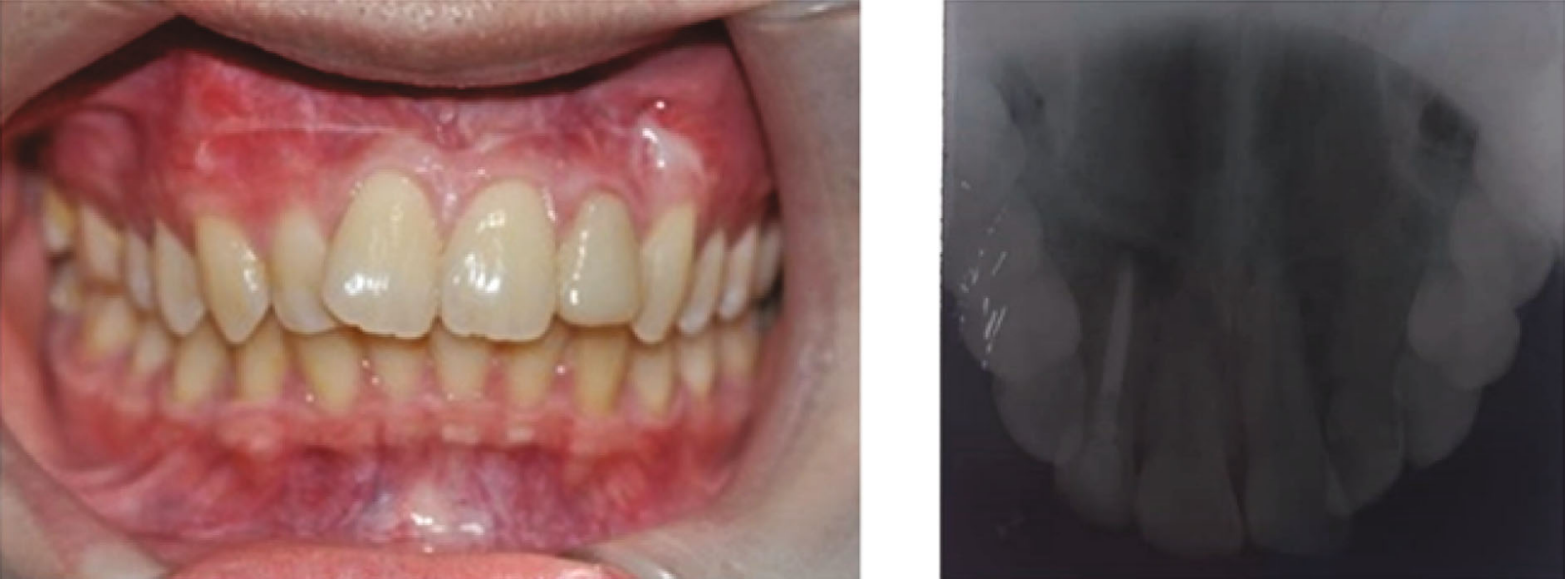
One year follow-up in radiograph with patient's tooth color appearance.

The patient came back after 2 weeks for an indirect full veneer preparation. Minimal enamel reduction was constructed and an impression taken with light and heavy body hydrophilic vinyl polysiloxane material (Examix™ NDS, Japan). Laboratory construction for IPS E-max ceramic (Ivoclar Vivadent, Liechtenstein) was completed after 3 days and the patient was called for full veneer issue. The tooth was adequately isolated by the placement of a rubber dam, and RelyX™ Unicem self-adhesive universal resin cement (3M ESPE, Germany) was used for the cementation. One-year follow-up showed an almost complete healing of the lesion, and acceptable tooth color appearance (Figure 7).

## 3. Discussion

In the present clinical cases, the quality of the coronal seal and root canal filling was not achieved to a satisfactory standard. The extruded gutta percha and the presence of bacterial colonies in the periapical area could be major contribution factors to the inflammatory periapical lesion. Even though the gutta percha for the root canal filling proved to be least toxic and compatible with connective tissue, the studies also revealed that it might cause an early short-term inflammatory tissue reaction [5, 6]. Nonsurgical root canal retreatment is always the first line approach to deal with a failure root canal-treated tooth. In the present clinical cases, nonsurgical retreatment was performed to clean and disinfect the defective canal and to retract the overfilling gutta percha beyond the apical end of the root. Since the patient had suffered for a very long time from the inflammatory lesion and there was still no sign of healing after foreign body removal, we decided to proceed with periapical surgery. The surgery itself is always the last resort, with the main objective of removing the disease of periapical tissues. It is also aimed at sealing the apical root canal system with biocompatible materials in order to facilitate the regeneration of hard and soft tissues, including the formation of new attachment cells [7]. Furthermore, all the endodontic surgery should be undertaken in order for a histopathology assessment to confirm the diagnosis [8]. In the present clinical cases, histopathological biopsy had revealed that the lesion contained condensed fibrous connective tissue which was infiltrated by some hemorrhage and moderate chronic inflammatory cells comprising polymorphonuclear neutrophils, lymphoplasma cells, and macrophages with no epithelium lining detected. Therefore, the diagnosis was confirmed as periapical granuloma. Studies showed that the periapical granuloma contains high inflammatory cell and mediators such as Cyclooxygenase-2 (COX-2) and Tumor Necrosis Factor Alpha (TNF-*α*). Cytokines Interleukin-33 (IL-33) which belongs to the Interleukin-1 (IL-1) family and its receptor-related ST2 were also present and involved in periapical inflammation [9, 10]. A study revealed that the secretion of TNF-*α* by macrophages and of COX-2 by several cells was higher in periapical granuloma indicating a greater inflammatory response in these lesions [9]. Furthermore, another study showed increased numbers of IL-33- and ST2-positive fibroblasts in periapical lesions when compared to healthy periapical tissues suggesting that IL-33/ST2 signaling may be involved in periapical inflammation and tissue fibrosis [10]. The formation of new bone had been observed as early as 12 weeks after periapical surgical repair and placement of substitute bone [11]. The use of hydroxyapatite (HA) to fill the osseous defect has been proven to produce a very successful result. HA granules act as a scaffold for blood vessels and migration of osteoblast from surrounding healthy bone, thus promoting differentiation of mesenchymal cells into cartilages and bone. HA will be slowly resorbed and later will be replaced by new bone formation [12, 13]. In our case, freeze-dried cancellous Osteo-Lamb bone graft was used as it was well accepted by the patient and never contradicted with his faith and beliefs. Because of the small anatomical and minimal sound tooth structure, we decided to proceed with a less invasive tooth preparation for the discolored tooth appearance. Nonvital internal bleaching was first constructed for the tooth to achieve acceptable color appearance before proceeding with the indirect full veneer restoration. E-max veneers were chosen since they are made up of porcelain which is highly resistant to stain and only minimal enamel reduction is needed to fit a 0.3 mm thickness of the material [14, 15].

## 4. Conclusion

A multidisciplinary approach is necessary for a tooth with a previous failure root canal treatment, where consideration for the patient's concern is always prioritized. This case presented with a successful outcome of nonsurgical and surgical root canal retreatment followed by internal bleaching and finally with indirect ceramic veneer restoration.
